# Mechanisms Regulating the UPS-ALS Crosstalk: The Role of Proteaphagy

**DOI:** 10.3390/molecules25102352

**Published:** 2020-05-18

**Authors:** Grégoire Quinet, Maria Gonzalez-Santamarta, Clara Louche, Manuel S. Rodriguez

**Affiliations:** ITAV-CNRS USR 3505 IPBS-UPS, 1 Place Pierre Potier, 31106 Toulouse, France; Gregoire.QUINET@itav.fr (G.Q.); Maria.GONZALEZ-SANTAMARTA@itav.fr (M.G.-S.); clara.louche@itav.fr (C.L.)

**Keywords:** ubiquitin proteasome system, autophagy, ubiquitin-like, proteaphagy, pathology

## Abstract

Protein degradation is tightly regulated inside cells because of its utmost importance for protein homeostasis (proteostasis). The two major intracellular proteolytic pathways are the ubiquitin-proteasome and the autophagy-lysosome systems which ensure the fate of proteins when modified by various members of the ubiquitin family. These pathways are tightly interconnected by receptors and cofactors that recognize distinct chain architectures to connect with either the proteasome or autophagy under distinct physiologic and pathologic situations. The degradation of proteasome by autophagy, known as proteaphagy, plays an important role in this crosstalk since it favours the activity of autophagy in the absence of fully active proteasomes. Recently described in several biological models, proteaphagy appears to help the cell to survive when proteostasis is broken by the absence of nutrients or the excess of proteins accumulated under various stress conditions. Emerging evidence indicates that proteaphagy could be permanently activated in some types of cancer or when chemoresistance is observed in patients.

## 1. Introduction

In order to maintain cell viability, protein homeostasis (proteostasis) needs to be tightly controlled. Proteostasis is regulated by many mechanisms, including various post-translational modifications (PTM). PTMs can affect the half-life of proteins, their localization, their activity or even their interactions with other cellular components. Protein PTM are highly dynamic and controlled by the action of modifying/de-modifying enzymes that attach/detach small chemical groups, peptides or even complex molecules. The best-known protein modification by a small polypeptide is ubiquitin (Ub), which together with other ubiquitin-like (UbL) proteins integrates a family sharing the same β-grasp folding [1]. Ubiquitin family members are about 10 to 20 kDa in size when free. The C-terminal glycine residues of Ub or UbL moieties are attached or conjugated to a lysine residue (in most cases) on the substrate protein. The conjugation of Ub and UbL to protein substrates is mediated by a three-step enzymatic thiol-ester cascade that involves distinct sets of enzymes. A first ATP-dependent step occurs with the action of an E1 activating enzyme. The Ub or UbL moiety is then transferred to an E2 conjugating enzyme, to finally be specifically attached to a protein substrate with the help of an E3 ligase [2]. As mentioned, those modifications are reversible and each modification can be cleaved by specific enzymes such as deubiquitylating (DUB) [3], SUMO specific proteases (SUSPs) [4] or deNEDDylating (NEDP1) enzymes [5], among other isopeptidases.

The ubiquitin proteasome system (UPS) and the autophagy lysosome system (ALS) are the two major intracellular proteolytic machineries in eukaryotic cells [6]. The UPS system relies on the polyubiquitylation of target proteins and their subsequent degradation by the proteasome [7,8]. While ubiquitylation also contributes to recruiting cargoes that are targeted to the ALS for degradation, the chain topology appears to be different to the one recognized by the proteasome. In particular, the ALS drives the degradation of cellular components, such as organelles, but also large protein aggregates. Selective autophagy events (macroautophagy) require the formation of autophagosomes that will fuse with lysosomes to degrade sequestered components [9].

PTMs contribute to coordinating the action of the UPS and ALS under distinct cellular stress situations such as multiple infections, inflammations, degenerative diseases, cancers or in response to medical treatments. This review presents some of the major roles of the UPS and ALS in protein homeostasis and the cooperation mechanisms activated in various pathologic situations that help the cell to cope with a disrupted proteostasis. Recent findings on proteaphagy will be discussed. In particular, its role in the ALS–UPS exchanges under nutrient starvation or after proteasome inhibition.

## 2. The Ubiquitin Proteasome System (UPS)

The ubiquitin proteasome system (UPS) is one of the main mechanisms for intracellular protein degradation and is essential to maintain protein homeostasis. This proteolytic mechanism is tightly controlled, to ensure the correct turnover of substrates involved in a wide variety of cellular processes. Ubiquitylated cargoes are recognized by proteasome subunits and cofactors that present substrates to the catalytic core of the proteasome [7,10]. Protein ubiquitylation and degradation is tightly regulated by a large number of cellular factors including Ub and UbL, enzymes involved in the modification/de-modification of substrates, chaperones and permanent or transient components of the proteasome [11,12].

### 2.1. The 20S Proteasome

The proteasome is a large complex multidomain protein (2.5 MDa) that drives the degradation of short-lived proteins in need of accurate turnover control. Proteasomes are found in nuclear and cytosolic compartments, associated or not to organelles [13].

**Figure 1:**
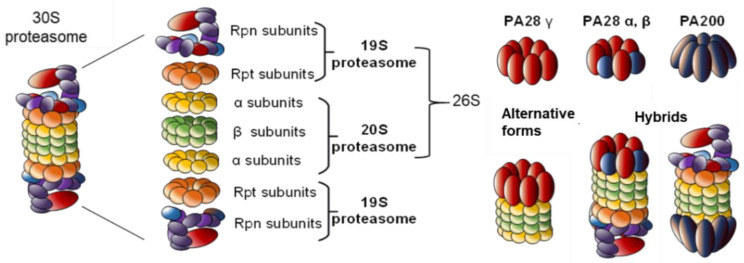
Proteasome complexity. The 26S proteasome is the most studied proteasome. However, alternative regulatory particles 19S, PA28 or PA200 are able to cap the core 20S proteasome to modulate its proteolytic activity. All these proteasome complexes (20S, 26S, 30S or hybrids) have been found in cells.

The core particle (CP) of this barrel-shaped complex is also known as the 20S proteasome. It stacks two outer rings composed of seven α-subunits (α1–7) and two inner rings of seven β-subunits (β1–7) (Figure 1). The catalytic sites within the proteolytic chamber are present in three β subunits (β1, β2 and β5) and respectively display trypsin-like, post–glutamyl peptide hydrolase–like and chymotrypsin-like activities. Structural studies revealed that α-rings form a closed structure which prevents proteins from entering the inner chamber of the β-rings [14]. Narrow axial pores of the 20S barrel allow access to this catalytic chamber so that only unfolded proteins can enter for degradation [15]. Although 20S alone are found in cells and degrade unstructured substrates in the absence of ubiquitylation [16], their association with 19S tightly controls the specific degradation of ubiquitin modified substrates [10].

Cytokines that activate immune response can induce several isoforms of 20S subunits. The genes encode for inducible proteasomal β subunits, so-called β1i, β2i and β5i. These subunits replace their counterparts in the constitutive 20S, building the immunoproteasome [17]. This alternative form of 20S proteasome has different substrate selectivity compared to the normal proteasome. In particular, it is essential for the MHC-I antigen processing, since its degradative action is at the origin of antigenic peptides [18]. The thymoproteasome has also been described as an alternative form of the CP. A β5t expressed specifically in the thymus is incorporated into the 20S, together with β1i and β2i subunits. This thymoproteasome has a specific role in thymic positive selection of CD8+ T cells [19].

### 2.2. The 19S Complex

Proteasomes exist in several complexes, depending on the association between a CP and one or two regulatory particles (RP). The CP associated to the RP 19S is the most abundant and best characterized form of cellular proteasome, the so-called 26S proteasome [14]. The 19S, also known as P700 complex, not only recognizes and tethers targeted polyubiquitylated proteins but also removes ubiquitin chains [20], unfolds protein substrates [21], opens the closed α-ring [22], and allow unfolded proteins to enter into the CP for proteolysis. The 19S caps one or both ends of the CP, forming respectively the 26S or the 30S proteasomes (Figure 1). A base and a lid subcomplexes form the 19S [10]. At least nine non-ATPase (RPN) subunits form the lid complex: RPN3, RPN5, RPN6, RPN7, RPN8, RPN9, RPN11, RPN12 and RPN15. The base of the 19S complex includes four non-ATPase subunits (RPN1, RPN2, RPN10 and RPN13) and six homologous AAA + ATPase subunits (RPT1–RPT6) [10]. The base has various roles including the opening of the channel of the α-ring. The six ATPase subunits of the base form a hexameric ring that controls the entry of substrates into the catalytic sites of the CP [15]. RPN subunits have an important structural role, since recent evidence revealed that these subunits contribute to complex conformational changes to process specific substrates [15,23]. For instance, RPN1 and RPN2 work together to create a surface for substrate recruitment and interaction with the 20S [24]. These regulatory particles also ensure deubiquitylation of protein substrates by the action of several DUBs. While RPN11 is essential for RP-CP assembly [25], this subunit also cleaves polyubiquitin chains at a proximal site and contributes to recycling ubiquitin chains [26,27]. The C-terminal domain of RPN13 also has a DUB activity that optimises the cleavage of ubiquitin chains when close to the proteasome. Other DUBs cleave at distal sites including Usp14 anchored to RPN1 [12,14] or Uch37 associated to RPN2, bound itself to RPN13 [28].

The 19S contains several ubiquitin receptors to efficiently capture ubiquitylated proteins. The docking and the recognition of ubiquitylated substrates is performed through ubiquitin binding domains (UBD), located either within intrinsic proteasomal ubiquitin receptors or extrinsic ubiquitin receptors. The three intrinsic ubiquitin receptors of the 19S are RPN1, RPN10 and RPN13. The Ub-interacting motif (UIM) located at the C-terminus of RPN10 binds selectively monoubiquitin but also K48 and K63 polyubiquitin chains [29]. Ubiquitylation of RPN10 modulates substrate recruitment to proteasome [30] and its association with the 19S [31]. RPN13 binds di-ubiquitin through a pleckstrin-like receptor for ubiquitin (Pru) domain located at its N-terminus [32]. RPN1 carries a UBD that binds both ubiquitin and UbL proteins [33]. RPT5 also binds polyubiquitylated proteins in vitro, but in vivo evidence is still lacking [34]. Several extrinsic ubiquitin receptors such as Rad23, Dsk2, Ddi1 and Sem1 contain in their structure both UbL and UBA (ubiquitin-associated) domains. Their UBA domains interact with a specific ubiquitin signal, while their UbL domains allow interaction with proteasome subunits [35,36]. In particular, the UBD of RPN1 interacts with the UbL domain of Rad23 and triggers its tethering to proteasome [24,37]. Other ubiquitin receptors include VCP (AKA Cdc48 or p97 ATPase) that binds proteasome and polyubiquitylated substrates. This highly conserved AAA + ATPase is essential for proteasomal degradation of well-folded proteins. VCP carries a protein-unfoldase activity that is able to extract ubiquitylated proteins from complexes, unfolds the target to finally ensure its processing into the catalytic core of the 26S proteasome [38].

### 2.3. Other CP Regulators

The CP can also be regulated by several RP such as PA28 (AKA 11S or REG) and PA200 (AKA Blm10), two complexes with distinct characteristics and biological roles (Figure 1). Three structurally related PA28 proteins known as α, β and γ share around 50% of homology. While PA28α and PA28β assembled into hetero-oligomeric complexes with alternating α and β subunits, PA28γ forms homopolymers [39]. PA28α and PA28β are located in the cytoplasm, whereas PA28γ is mainly located in the nucleus and in perinuclear areas. PA28α/β complexes activate peptidase activities of the 20S proteasome and contribute to the production of cytotoxic T lymphocyte (CTL) epitopes [18]. Some evidence indicates that PA28γ functions as a regulator of cell proliferation and body growth in mice. In PA28γ^−/−^ mice, neither PA28α nor PA28β compensate for PA28γ deficiency, suggesting their non-redundant roles [40]. PA28γ is implicated in various functions such as DNA damage response, apoptosis signalling, or transcriptional regulation of metabolism: it is involved in both ubiquitin-dependent and -independent recognition of substrates, to mediate proteasome-mediated protein turnover [41]. In yeast, PA200 has been shown to regulate proteasome assembly, maturation and/or proteolytic activity. This regulatory particle binds to the 20S but it is also found in hybrid proteasomes together with the 19S [14,42] (Figure 1).

### 2.4. The Hybrid Proteasome

Hybrid proteasomes are composed of two distinct regulators capping both ends of the CP. They contribute to an efficient coordination of cell proteolysis. For instance, PA28 and 19S can simultaneously bind to the two extremes of the 20S particle, forming the 19S–20S–PA28 “hybrid proteasome” complex (Figure 1) [39,43]. The RP first recognizes protein substrates to be internalized into the cavity of the 20S with an enhanced cleavage activity when bound to the PA28 complex. PA28 has been shown to activate the ATP-dependent degradation of ornithine decarboxylase (ODC) even in the absence of ubiquitylation but in the presence of antizyme (ODC inhibitor) [43]. Hybrid proteasomes also enhance the hydrolysis of small peptides that are different from those typically processed by the 26S proteasome. This has been associated to the capacity of PA28 to enhance antigen presentation. Interestingly, IFN-γ enhances the expression of the PA28αβ complex, favoring the formation of a hybrid proteasome and justifying its role in the processing of intracellular antigens [44].

All of these proteasomes create a large variety of complexes that can adapt for an optimized intracellular proteolysis activity. Proteasomes play major roles to face cellular stresses, and their dysregulation causes many pathologies, including neurodegenerative and cardiovascular disorders, respiratory diseases, and cancers [45]. Therefore, efficient proteasome activity is essential to maintain proteostasis in healthy cells.

## 3. Autophagy Lysosome System (ALS)

The autophagy lysosome system (ALS) is, together with the UPS, one of the main intracellular degradation systems. Literally meaning self-eating in Greek, autophagy digests long-lived, protein aggregates, stress RNA granules, and abnormal cytoplasmic organelles, including mitochondria, among others. Three types of autophagy have been identified so far: the chaperone-mediated autophagy, the microautophagy and the macroautophagy. The chaperone-mediated autophagy uses HSC70 to sequester into lysosomes proteins containing a KFERQ motif. Microautophagy is a direct lysosomal uptaking of substrates, whereas during macroautophagy (here referred as autophagy), cytosolic cargos are sequestered by a double membrane vesicle within which a portion of the cytoplasm is trapped in a complex multistep process. A tight regulation of autophagy leads to the selective degradation of substrates by the lysosome [9]. Therefore, the ALS plays a wide range of physiological and pathological roles.

### 3.1. Basal and Induced Autophagy

Early studies reported autophagy as a non-selective pathway, in which cargoes were randomly degraded. Indeed, autophagy was initially considered as a bulk degradation pathway, activated during nutrient deprivation, to ensure recycling of building blocks [46]. However, growing evidence supports the existence of an intricate selective process that contributes to intracellular homeostasis in non-starved cells [47]. Only one selective autophagy pathway can exclusively recognize and eliminate particular structures that must be degraded [46]. Hence, mitophagy drives mitochondria degradation, xenophagy targets pathogens, aggrephagy destroys aggregates, proteaphagy degrades proteasomes, etc. Different regulators modulate the selectivity of autophagy, including ubiquitin, which serves as a major degradation signal for this pathway [48]. However, a variety of cargoes are committed selectively to autophagy, in an ubiquitin-independent manner [49].

**Figure 2:**
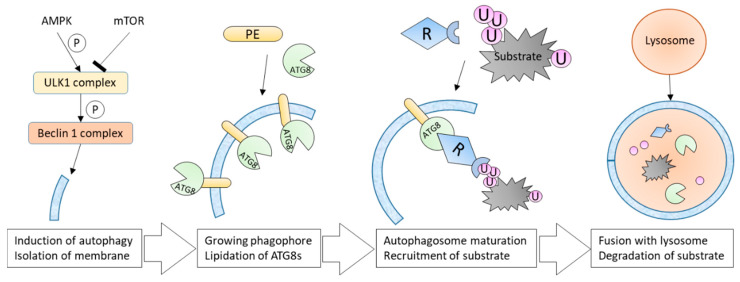
Selective autophagy pathways ensure the degradation of specific cargoes but also bulk of proteins. A complex signaling pathway regulates autophagy and induces the lipidation of ATG8 proteins within the newly formed cup shaped membrane, called phagophore. Recruitment of the autophagy substrate marks the maturation of the autophagosome. The substrate tethering to lipidated ATG8 is ensured by autophagy receptors (R). Autophagosomes then fuse with lysosomes, forming autolysosomes in which trapped substrates are degraded.

Basal level autophagy runs continuously under normal conditions [47], but stresses such as starvation, multiple infections, heat or drug treatment can strongly activate the process. The signalling pathways involved in autophagy regulation are centralized around the ULK1 (for unc-51 like autophagy activating kinase 1, also called ATG1) and Beclin1 complexes (Figure 2). ULK1 and Beclin1 phosphorylation is modulated by signalling proteins such as mTOR, Akt, AMPK and other PKA kinases. These phosphorylation steps regulate the initiation of the autophagic machinery [50]. The induction of autophagy triggers the isolation and elongation of a large cup-shaped double membrane that forms the growing phagophore (Figure 2). Although the origin of this membrane is not clear, various intracellular sources have been proposed, such as the endoplasmic reticulum (ER), the Golgi complex or the mitochondria [51]. Around 20 AuTophaGy (ATG) genes mediate the autophagy process [52]. These proteins are recruited to the phagophore and regulate the later autophagosome formation and maturation. During autophagy induction, ATG8 proteins are modified by phosphatidylethanolamine (PE). The lipidated ATG8 proteins localize at both sides of the phagophore, controlling the size of the future autophagosome [53,54]. Autophagosome maturation and the subsequent recruitment of substrates driven by ATG proteins (see below) lead to the late fusion with either vacuoles, endosomes or lysosomes (Figure 2). The autolysosome formed degrades targeted substrates using a series of lysosomal/vacuolar acid hydrolases such as cathepsins in mammalian cells. These degradation events produce small molecules such as amino acids that are transported back in the cytosol for recycling [55,56].

### 3.2. ATG8 Proteins Family, Characteristics and Functions

ATG8s are central components of autophagy regulation because of their essential role in autophagosome formation and maturation. This protein family is composed of six members in mammalian cells, LC3A, B and C, GABARAP, GABARAP L1 and GABARAP L2. The β grasp folding of ubiquitin is well conserved among the members of the ATG8 family and plays an essential role in protein–protein interactions. However, ATG8 proteins contain a supplementary hydrophobic pocket that engages interaction networks with autophagy and membrane trafficking components [57]. During the autophagy process, LC3/GABARAP proteins are conjugated to PE in an enzymatic manner. The mature form of ATG8, which will be covalently linked to PE, is generated through the processing of a high molecular weight precursor that is cleaved by the protease ATG4 [58]. To get conjugated to PE, ATG8 has to be first activated by ATG7 (E1). The conjugation requires the action of ATG3 (E2) and a mature E3 complex integrated by ATG5, ATG12 and ATG16. The integration of this E3 complex is mediated by ATG10 (E2) that covalently links ATG12 and ATG5 [59]. Since PE is the second-most abundant phospholipid found in biological membranes in mammals, its abundance positively regulates autophagy [60]. PE functions as an anchor of ATG8 to autophagosome membranes. ATG8s integration to phagophore through PE mediates the membrane fusion needed for a proper autophagosome formation [48,61].

A recent study showed that blocking the ATG8 conjugation system by knocking out ATG5 or ATG7 dramatically reduces membrane elongation and closure of autophagosomes, although autophagosome formation is not fully abolished [52]. Although Nguyen et al. showed that LC3/GABARAP proteins are not essential during the autophagosome formation, they appear to be crucial for the autophagosome-lysosome fusion [62]. Sometimes considered as proteins with common or redundant functions, the members of the LC3/GABARAP family play specialized and distinct roles. As an example, a recent study reveals a striking opposite role of LC3B/C and GABARAP/GABARAPL1 in autophagy induction. Even if LC3s or GABARAPs binds to the autophagy activation complex ULK1, they respectively trigger a negative or a positive regulation of autophagy [63]. Furthermore, LC3/GABARAP proteins play a pivotal role in selective autophagy by ensuring the docking of specific substrates to autophagosome membranes. LC3/GABARAP proteins act in non-redundant ways together with a large variety of autophagy receptors to tether specific targets for degradation. For instance, the presence of LC3C in autophagosome membranes is crucial for innate immunity during bacterial infection. Its interaction with the autophagy receptor nuclear domain 10 protein 52 (NDP52) drives an antibacterial autophagy, also called xenophagy, that protects host cytoplasm against *Salmonella enterica* [64].

### 3.3. Autophagy Receptors

The presentation of cargoes to the ALS is driven by more than 30 autophagy receptors, also called sequestosome-1-like receptors (SLRs) after the first described p62/SQSTM1 (sequestosome 1) [65]. Other well studied SLRs include njext to BRCA1 gene 1 protein (NBR1), optineurin (OPTN) or NDP52, that share common functional domains (Table 1). The diversity of receptors underlines the complex regulation of selective autophagy mechanisms. Some of them show functional redundancy for cargo recognition and cooperate with cofactors [66]. Furthermore, SLRs can be involved in both ubiquitin-dependent and -independent mechanisms of autophagy degradation [49].

The human cargo receptor p62 mediates the degradation of many substrates, such as aggregated proteins or cytosolic bacteria. P62 carries a ubiquitin-associated domain (UBA), a UBD that binds ubiquitylated cargoes. The UBA domain of p62 can homodimerize, modulating its interaction with monoubiquitin [9]. P62 and other autophagy receptors contain LC3 interacting regions (LIR). This affinity domain interacts with ATG8 proteins tethered into the autophagosome membrane (Table 1). Furthermore, p62 display a N-Terminal PB1 domain that triggers its polymerization. The scaffolding of these PB1 domains forms helical filaments of p62 polymers of variable lengths, that interact with both ATG8s and long ubiquitin chains enhancing their interactions. These large polymers of p62 provide a large molecular scaffold for autophagosome and ensure ubiquitylated cargo recruitment to structures where ATG8 proteins are lipidated [68]. Interestingly, oligomeric p62 preferentially binds to linear and K63-linked ubiquitin chains, compared to K48-linked chains. For this reason, K63 ubiquitin chains are commonly admitted as the signal for autophagy degradation. In contrast, K48 ubiquitin chains have been proposed to disrupt p62 oligomers, suggesting that they are not the preferred targets for p62-mediated degradation in vivo [76]. p62 has also been shown to drive cargoes to autophagosomes, in a ubiquitin-independent manner. A protein that needs to be degraded can get its N-terminus cleaved or modified by PTMs, building a signal for degradation called N-degron. Through its ZZ domain, p62 is able to bind selectively N-degrons, oligomerize and deliver these cargoes to autophagosomes [77].

NBR1 is another autophagy receptor carrying UBA, LIR and PB1 domains, with similar folding but distinct amino-acid sequence to the ones of p62. NBR1 plays an essential role in peroxisomes autophagy and works as a specific autophagy receptor for these organelles [66] (Table 1). There is no evidence supporting the idea that the PB1 domain of NBR1 is able to form polymers. However, it has been shown that NBR1 can form hetero-oligomers with p62 and cooperate in autophagy degradation [68]. OPTN acts as an autophagy receptor during xenophagy, mitophagy, as well as in aggrephagy. Considered as ancestral bacteria, mitochondria (mitophagy) uses the same receptor as xenophagy. However, it is clear that distinct signalling pathways and mechanisms activate and regulate these selective autophagy events. In mitophagy, OPTN is recruited to damaged mitochondria along with NDP52, therefore both receptors play redundant roles [78]. Interestingly, mitophagy and xenophagy also involve OPTN and p62, but both receptors lead to independent degradation in separated autophagosomes [79]. This evidence suggests a strong collaboration between autophagy receptors to regulate specificity of lysosome degradation [79] (Table 1). Furthermore, post-translational modifications of autophagy receptors can modulate their activity, adding another layer of complexity in the regulation of selective autophagy events. As an example, the OPTN phosphorylation on serine −177 located upstream of the LIR motif regulates its interaction with ATG8 proteins and is needed for the autophagy of *Salmonella enterica* during infection [70].

### 3.4. The Role of LIR Motifs

The LIR motif is a small peptide sequence that has affinity for ATG8 proteins. LIR-containing proteins can be autophagy receptors but also members of basal autophagy regulation, vesicle-associated proteins, and specific signalling proteins. The great number of possible LIR sequences are gathered in three consensuses and named depending of the first amino acid of hydrophobic core sequence: tryptophan, phenylalanine or tyrosine. Birgisdottir et al. defined the possible core consensus sequences as [W/F/Y]xx[L/I/V], and displayed variable binding affinities for the hydrophobic pockets of the different LC3/GABARAP proteins. As an example, the structure of p62 LIR motif reveals a W-x-x-L motif that gives a non-exclusive preference for LC3B proteins. OPTN carries the F-x-x-I consensus while NBR1 displays the less common Y-x-x-I consensus [80]. The LIR motif from OPTN shows stronger interaction with GABARAPs than with LC3s [81], although the affinity switches toward LC3B when phosphorylated at S177 by the TANK binding kinase (TBK1) [69].

Non-canonical LIR motifs have also been reported, such as the SKIP carboxyl homology (SKICH) domain of the autophagy receptor NDP52. This domain is essential for selective interaction with LC3C during *Salmonella* infections to drive the antibacterial autophagy [64]. In addition to the four amino acids of the LIR motif, other adjacent amino acid residues are needed for specificity and affinity toward LC3/GABARAP proteins. For example, the importance of the acidic aspartic residues N-terminal to the LIR motif of p62 was verified by alanine substitutions [82]. Therefore, differences in the amino acids surrounding LIR motifs create different autophagy receptors affinities for LC3/GABARAP proteins.

To sum up, in order to explore the complexity of selective autophagy regulation, one should first consider the ubiquitin-chains present on the tagged substrates and the nature of the UBDs carried by dozens of autophagy receptors. Second, members of the LC3/GABARAP family interact with autophagy receptors through a diversity of LIR sequences to sort and tether autophagy substrates toward autophagosome membranes. Furthermore, homo and hetero polymerizations of autophagy receptors modulate their interactions with ubiquitin signals and/or LC3/GABARAP proteins. Actions of several autophagy receptors during one selective autophagy event can occur, with or without collaboration with other receptors. Finally, PTMs occurring on all these proteins can modulate their activities as well. The number of possible combinations involved in cargo recognition reveals the level of complexity of selective autophagy processes in mammalian cells.

## 4. Crosstalk between ALS and UPS

Although the UPS and the ALS have been initially considered to be independent proteolytic mechanisms, their interconnection has been supported by increasing evidence. The level of similarity and overlapping of regulatory components in these two pathways supports the notion that they belong to a single coordinated proteolytic network [83]. In order to adapt to the changing cellular environment, in particular during pathologies, a highly regulated network of molecular mechanisms modulates an efficient crosstalk. The following section will review some of the most important mechanisms involved in the regulation of the interconnection between the UPS and the ALS [83,84,85].

### 4.1. Central Role of Ubiquitin in UPS/ALS Crosstalk

Ubiquitin chain architecture appears to be determinant to drive protein substrates to one or the other proteolytic pathway. In addition to K48, K11 and K29 ubiquitin linkages appear to contribute to UPS degradation [86]. In contrast, K63 and K6 ubiquitin linkages have been considered as autophagy degradation signals [87]. Other atypical ubiquitin linkages have been proposed as signals for autophagy degradation, although little is known about their specific mechanism. One example is the Met1 linear ubiquitin chain that acts as a signal for autophagy degradation during bacterial xenophagy [83]. Although p62 protein shows a higher binding affinity for K63 ubiquitin linkages, it can recognize K48 chains as well. A competition for K48-linked ubiquitin chains between p62 and p97/VCP (ubiquitin-binding ER-associated degradation protein) determines the degradation pathway to be taken by protein substrates [86]. Most likely, one ubiquitin linkage will not be enough to determine protein degradation since multiple ubiquitin chain topologies are involved in proteolysis regulation. Furthermore, other post-translational modifications including acetylation, phosphorylation or various UbL proteins would directly affect chain composition with an impact on proteolysis. This large panel of chain possibilities and complexity integrated under the name of “ubiquitin code”, is implicated in the final proteolytic decision [83,88]. More information on the role of ubiquitin chains in the UPS/ALS was nicely gathered in recent reviews [84,85].

E3 ligases can also act as key elements to regulate the connection between UPS and ALS, as they build multiple ubiquitin chains but can also be targets of the UPS and ALS. One example is the E3 ligase E124 that is responsible for targeting ubiquitin ligases for degradation by the ALS. It has been proved that E124 promotes the degradation of several E3 enzymes (most of them belonging to the RING type), such as TRAF2, RINCK2 and several Tripartite Motif (TRIM) family enzymes: TRIM28, TRIM21 and TRIM1 among others. Interestingly, TRIM family proteins have been recently shown to act as autophagy receptors and regulators of the autophagosome formation [89]. The biological implications of the degradation of RING ubiquitin ligases and its impact on the regulation of the autophagy system are not well understood [85]. Some of these E3 ligases control factors activating transcription that promotes autophagy initiation such as the previously mentioned NF-κB or FOXOs [90]_._

The N-end rule pathway is a proteolytic system in which single amino acids in the N-terminal part of proteins act as signals for degradation (N-degrons). In eukaryotes there are two different pathways. The Ac/N-end rule pathway targets proteins containing N(α)-terminally acetylated (Nt-acetylated) residues. The Arg/N-end rule pathway recognizes unacetylated N-terminal residues and involves N-terminal arginylation. N-terminal arginylated degrons are recognized by UBR box family proteins (UBR1, UBR2, UBR4, UBR5), that promote ubiquitylation to mediate the degradation by the proteasome. Recently, SQSTM1/p62 protein was identified to be a N-recognin that binds N-Arg and other N-degrons (Type 1 and 2). P62 hence mediates autophagic degradation of ER-residing molecular chaperones and their associated protein cargoes [77]. The N-end rule pathway regulates autophagy degradation by limiting the participation of p62 to selective autophagy events. In this context, cellular proteotoxic stress developed after the accumulation of misfolded ER proteins promotes the N-terminal argynilation of cytosolic chaperones like BiP. Argynilated chaperons are recognized by the ZZ domain of p62 and oligomerized. Aggregated p62/chaperons interact with LC3 and are targeted to a ubiquitin-independent mediated autophagy since the UBD of p62 is not involved in this process [83].

### 4.2. Compensatory UPS-ALS Mechanisms

Some of the strongest evidence of the UPS–ALS interconnection was revealed after the chemical or genetic inactivation of the proteasome that results in the activation of autophagy [91]. Different proteasome inhibitors used in clinic, such as bortezomib (BTZ) or NP-0052, have been reported to activate autophagy, relieving cells from cellular proteotoxicity after protein accumulation [92]. How this compensatory mechanism is regulated is not well understood. However different mechanisms have been proposed, including a role for the N-end rule pathway, the UPS-ER-autophagy circuit or the tumour suppressor protein p53 [93,94,95].

Various transcription factors have been shown to connect the UPS and the ALS, including processes mediated by p53. p53 is one of the best characterized targets of the UPS that plays a dual role in autophagy, depending on its cellular localization. In the nucleus, p53 acts as a transcription factor for autophagy-related genes such as ATG2, ATG4, ATG7, and ULK1, known to activate autophagy [96]. Under starvation and proteasome impairment, cytosolic p53 leads to the activation of the AMPK, that in turn inhibits the mTOR pathway, leading to autophagy activation [83,84,85,86,87,88,89,90,91,92,93,94,95,96,97]. Wang et al. revealed that the impairment of autophagy after knock down of ATG genes in colon cancer led to upregulation of proteasomal subunits β5 [98]. However, ATG5 and ATG7 knockouts in mice have been shown to accumulate ubiquitylated proteins in different tissues, although this compensatory mechanism could be influenced by the physio-pathology status of the cell [24,85]. Autophagy inhibition causes the accumulation of protein aggregates containing p62. These aggregates are thought to sequester proteasomal substrates but also positive regulators of the UPS, leading to a disruption in proteasomal flux [99].

The UPS has been a target for anti-cancer therapy for a couple of decades. However, resistance to proteasome inhibitors activate compensatory mechanisms, including a permanent activation of the ALS (see Section 5). Understanding how the ALS and the UPS communicate with each other is an actual challenge to find alternative treatments [85].

### 4.3. Other Mechanisms Impacting the UPS-ALS Crosstalk

After their synthesis, proteins are folded in the endoplasmic reticulum (ER). Misfolded proteins accumulated into the ER are retrotranslocated to the cytoplasm where they become targets for the UPS. This ER-associated degradation (ERAD) can be compensated by autophagy after ER stress. ER-stress upregulates Nrf2 target genes, which in turn induce autophagy [85]. In the UPS-ER-autophagy circuit, accumulated misfolded proteins promote the dissociation of the chaperon protein GRP78/BiP from the ER membrane. At the same time, different membrane receptors like PERK, IRE1α, ATF6α [94] activate ATG gene expression, as well as LC3 lipidation and autophagosome biogenesis [83].

Chaperone proteins such as C-terminus of HSP-70-interacting protein (CHIP) and BCL-2 associated athanogenes (BAG1 and 3) also determine the fate of protein degradation when misfolding events occur [85]. CHIP acts as a co-chaperone for Hsp70 and Hsp90, for the degradation of misfolded proteins through the addition of K48-linked ubiquitin chains [100]. However, CHIP can also mediate autophagy degradation by promoting K63 linkages. Associated CHIP-associated chaperones and E2 partners direct substrates to either proteasomal or autophagic degradation. Other chaperones involved are BAG1 and 3, that mediate proteasomal degradation. BAG3 interacts with Hsp70, CHIP and p62 for autophagy degradation. During aging, the BAG1/3 ratio is known to modulate autophagy activity [83].

Mitochondria and ER are the two cellular organelle sensors of reduced proteasome activity. The accumulation of proteins as a consequence of proteasome impairment results in an alteration of mitochondrial proteome, ROS generation, and induction of autophagy via AMPK activation [101]. As mitochondria senses the ATP status inside the cells, the cellular energy reserve is an important factor to regulate degradation. Parkin protein, a known E3 ligase, plays a critical role by mediating proteasomal degradation of mitochondrial substrates. At the same time, it has been reported that mitochondria can be a shuttling hub of misfolded proteins to be degraded by mitophagy when the UPS is overloaded [85].

## 5. Role of Proteaphagy in the UPS-ALS Crosstalk

**Figure 3:**
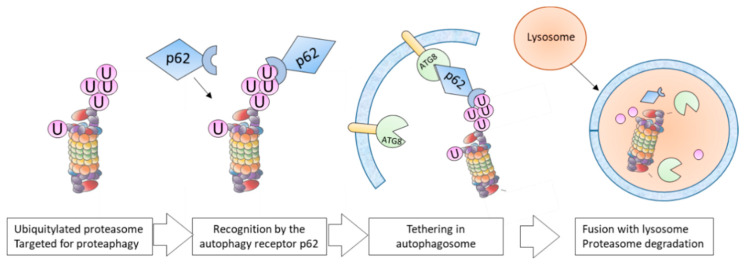
Main steps occurring during proteaphagy. In mammalian cells, ubiquitylated proteasomes are recognized by the autophagy receptor p62 that interacts with lipidated ATG8 proteins tethered into the autophagosome membranes. The late fusion with lysosome ensures the enzymatic degradation of captured proteasomes.

One of the most intriguing relationship between the UPS and autophagy is proteaphagy, that was demonstrated in distinct biological models (Figure 3). A restriction of nutrients or an accumulation of proteins disrupts proteostasis and activates this process. Importantly, permanently activated proteaphagy has been observed in certain human pathologies. This section sums up some of these recent findings.

### 5.1. Proteaphagy upon Nutrient Starvation

Upon nutrient starvation, autophagy is upregulated to face the lack of nitrogen, fixed carbon phosphate, zinc and other nutrients (Figure 3). In these situations, autophagy works as a recycling machine in order to recover the nutrient pool from dysfunctional cellular components [102,103]. During starvation, proteasomes might play a minor role in protein degradation because they are able to degrade one peptide at a time, with high energy cost. Nevertheless, proteasomes are relatively abundant, representing approximately 0.6% of total cell proteins [104]. Autophagy can be quickly induced during starvation and has a bulk protein degradation capacity. For this reason, the autophagic degradation of proteasomes can be seen as an efficient strategy to face nutrient limitation. Immuno-electron microscopy studies revealed the first hint of autophagic degradation of proteasomes, since proteasomes were observed in rat liver lysosomes upon starvation [105]. Later in time, quantitative proteomic analyses revealed proteasomal proteins among autophagosome-associated-proteins and regulators in basal and starvation-induced autophagy, suggesting that proteasomes are degraded in both conditions [106,107].

Recently, Marshall et al. proved that autophagic degradation of proteasomes occurs in a significant level in both *Arabidopsis taliana* and *Saccharomyces cerevisiae* under nitrogen starvation. In *Arabidopsis Thaliana*, 50% of proteasomes are degraded after 24 h while more than 80% of yeast proteasomes are degraded after 8 h of starvation [108,109]. This bulk autophagy of proteasomes activated under starvation was shown to be independent of autophagy receptors like RPN10 or NBR1, with no coincidence with proteasome ubiquitylation in plant model [108]. In yeast, no receptor that could drive proteasomes to autophagosomes during starvation has been identified [109,110]. This supports the hypothesis that bulk autophagy acts non-selectively without specific autophagy receptors. Nemec et al. revealed that in yeast, nitrogen starvation-induced proteaphagy involved the conserved nexins Snx4/41/42. The complete deletion of Snx4 impaired not only proteaphagy but also autophagy of fatty acid synthetase and ribosomes, indicating that Snx4 is a shared requirement for these selective autophagy events [111].

An important fraction of the proteasomes is located in the nucleus, whereas autophagy occurs in the cytoplasm. Can nuclear proteasomes or distinct proteasome complexes be impacted by proteaphagy? These are open questions for which only little evidence has been published. To be processed through bulk proteaphagy, nuclear proteasomes must be transported out of the nucleus. It is known that autophagy of nuclear components involves specific receptors such as ATG39 and ATG40 [112]. However, it has been excluded that ATG39 is required in nuclear proteasome autophagy upon starvation [108,110]. Some evidence supports a dissociation of CP and 19S prior to nuclear export, in order to allow cytosolic proteaphagy [110]. Waite et al. proposed a model in yeast where proteasomes are disassembled within the nucleus during nitrogen starvation and follow different routes toward autophagic degradation. They revealed that CP autophagy depends on the deubiquitylase Ubp3, while RP export for cytosolic degradation relies on ATG7 and ATG17 factors [110]. By artificially tethering the lid of the 19S proteasome to chromatin, Nemec et al. showed that a dissociation of 19S base and 20S is required prior to proteaphagy in nitrogen-starved yeast [111]. They proposed that proteasome dissociation attenuates CP activity when exported to cytosol, preventing interference with proteostasis in this compartment [111]. However, these mechanisms that ensure dissociation of nuclear proteasomes might not occur for cytosolic proteasomes.

In mammalian cells, proteaphagy is also activated in response to amino acid starvation. Cohen-Kaplan et al. showed a degradation of both CP and 19S RP subunits in starved HeLa cells. Interestingly, they revealed that proteaphagy induced by amino acid starvation is preceded by an increase in polyubiquitylation of proteasomes, mostly in RPN1, RPN2, RPN10 and RPN13 subunits. The autophagy receptor p62 mediates this degradation, making the bridge between lipidated LC3B and ubiquitylated proteasomes [113]. Altogether, these recent studies indicated that proteaphagy can be activated as part of the cellular response to starvation. This participation of distinct autophagy receptors in distinct biological models indicates that while this is a functionally conserved mechanism, the molecules involved can be distinct.

### 5.2. Proteaphagy of Non-Functional Proteasomes

The elimination of non-functional proteasomes by autophagy has been described in plants, yeast and mammalian cells (Figure 3). Both proteasome inhibitor treatment (bortezomib or MG132) and genetic alteration of proteasome subunits trigger proteaphagy. The extensive ubiquitylation of proteasomes, mainly on the 19S RP, has been highlighted in several studies [108,111]. The identities of modified subunits have not been fully characterized so far, but this ubiquitin signal is thought to mediate the recognition of impaired proteasomes by selective autophagy receptors and their clearance [114]. In *Arabidopsis Thaliana*, Marshall et al. proposed RPN10 to be the selective autophagy receptor for inactive 26S proteasomes. RPN10 was shown to bind on one hand ATG8 through a ubiquitin-interacting motif (UIM) and on the other hand ubiquitylated proteasomes. Interestingly, proteasome inhibitor-induced proteaphagy was hampered in RPN10 mutant, but starvation-induced proteaphagy was not affected. Furthermore, the deubiquitylase USP2 modulates RPN10 stability during proteasome inhibition, suggesting a central role of proteasome ubiquitylation in this context [108].

In yeast, separate routes are employed for proteaphagy during nitrogen starvation and proteasome inhibition. Proteasome inhibition induces ubiquitylation of proteasome prior to its degradation. Identification of autophagy receptors revealed Cue5 as a mediator of proteaphagy for both chemical inhibition and genetic mutation of 26S proteasomes. Co-immunoprecipitation assays supported that Cue5 tethers ubiquitylated 26S proteasomes to ATG8 [108]. Intriguingly, yeast proteaphagy likewise needs a prior aggregation of 26S proteasome into peri-vacuolar insoluble protein deposit (IPOD)-type structures. The IPOD formation upon proteasome inhibition suggests that there might be some overlap between proteaphagy and aggregaphagy, at least in a yeast model [79]. The chaperone Hsp42 has been shown to deliver dysfunctional proteasomes into IPODs, where extensive ubiquitylation might occur prior to proteaphagy [108]. In a *Dictyostelium discoideum* model, a recent study revealed direct interactions between RPN1 and RPN2 subunits with the core autophagosomal protein ATG16. In this organism, ATG16 appears to be required for RPN1 and RPN2 enrichment in ATG8a positive puncta, suggesting that ATG16 acts as proteaphagy adaptator [115].

It is still unclear whether proteaphagy induced by chemical inhibitors can drive degradation of CP and 19S RP proteasomes separately or target the 26S whole complex. Marshall et al. revealed that mutation in the α5 subunit triggers turnover of the CP only, and mutation in the RPN5 subunit triggers turnover of the 19S RP [108]. Specific ubiquitylation of proteasome subunits ensured by associated E3 ligases could play a crucial role in the regulation of proteaphagy. Furthermore, the involvement of distinct autophagy receptors in different biological models and in a stimuli-dependent manner could provide the quality control required to regulate this selective autophagy route.

## 6. Proteaphagy in Pathology

Proteaphagy has been shown to be induced by virulent *Pseudomonas syringae* pv tomato strain DC3000 during *Arabidopsis thaliana* infections. The bacterial protein T3E Hrp outer protein M1 (HopM1) works as a putative proteasome inhibitor to increase pathogenicity in plants [116], but also activates proteaphagy during infection [117] to escape host defenses. By enhancing autophagy flux and activating proteaphagy in host cells, *Pseudomonas syringae* suppresses proteasome function to promote virulence. This example is the only one demonstrating that proteaphagy can be manipulated by bacteria to enhance infection. Since it is known that several pathogenic bacteria modulate autophagy to escape their elimination through xenophagy (e.g., *Salmonella*, *Shigella*, *Legionella*, and *Mycobacterium*) [118], it is not excluded that proteaphagy could be part of the mechanisms employed by some other pathogens during human infection.

In yeast, nitrogen starvation or low ATP levels accumulate CP and RP separately in proteasome storage granules (PSG) [114]. Like IPODs, these PSG inclusions were proposed to serve as a proteasome protection mechanism from autophagy. Indeed, blocking delivery of proteasomes into PSGs induces their degradation by proteaphagy. It has thus been proposed that PSGs act as a reservoir of proteasomes for a rapid re-mobilization when proteolytic demand rises [114]. Protein aggregation and clustering, such as proteasome accumulation in PSGs or IPOD, are used by cells to survive under stress conditions. However, inappropriate regulation of cluster formation occurs and can be associated with diseases such as Alzheimer’s or Parkinson’s. Deregulation of mitophaghy is already associated with these neurodegenerative diseases. Loss of function of the ubiquitin ligase Parkin results in an accumulation of damaged mitochondria and an aggregation of proteins that can lead to neuronal death [118]. Therefore, investigating the link between proteaphagy and clustering regulation could ultimately improve the understanding of protein aggregation in neurodegenerative pathologies [119].

The proteasome inhibitor BTZ has been used to treat hematologic diseases such as multiple myeloma (MM) and mantle cell lymphoma (MCL), even if some patients do not respond or develop resistance to this treatment [120]. In acute myeloid leukaemia (AML), the proteasome inhibitor BTZ activates the autophagy degradation of crucial cellular factors when the FLT3-ITD translocation is present [91]. Proteaphagy has been recently observed in FLT3-ITD positive AML cells after BTZ treatment, and the chemical inhibition of autophagy and proteasome enhances apoptosis in those cells [121]. In MCL, proteaphagy has been easily observed in BTZ-resistant cells [122]. Proteasome subunits from both CP and RP are permanently degraded by autophagy in cells with acquired or innate BTZ resistance, in a p62-dependent manner. Interestingly, the more the cells are resistant to BTZ, the more proteaphagy seems to be activated. Pharmacological inhibition of autophagy by inhibitors such as bafilomycinA or chloroquine enhances BTZ-induced apoptosis in these cells [122]. Targeting autophagy to overcome BTZ resistance has already been proposed in these models, since it affects the stability of important cellular factors such as NOXA or NF-κB [93,94]. Importantly, the p62 inhibitor verteporfin (VTP) hampers proteaphagy in BTZ-resistant MCL cells. Thus, combining BTZ with autophagy inhibitors synergistically induces cytotoxic effects in BTZ-resistant cells and reduces tumor growth in xenografted animal models [122]. Targeting proteaphagy with pharmacological approaches can be considered as a relevant strategy to overcome BTZ-resistance in patients.

## 7. Conclusions

Maintaining the cellular proteostasis is of the upmost importance for cells health. The UPS and the ALS act in an orchestrated manner to guarantee the correct degradation of a large diversity of substrates such as proteins, aggregates, organelles or macromolecular complexes. Both systems are regulated through different signalling pathways, using different sensor molecules that will activate the most appropriated mechanisms to face aberrant situations. Ubiquitin and ubiquitin-like molecules play an important role in this crosstalk between the two major proteolysis pathways. No less important are the cellular factors and cofactors regulating these events or recognizing the degradation signals, like do the distinct autophagy receptors. Considering all possible combinations of signals and factors involved in the regulation of the UPS–ALS crosstalk, the plasticity of the cell to generate the appropriate response or to get adapted to a specific situation is wide and complex. Proteaphagy appears as an evolutionary conserved functional pathway by which organisms respond to an aberrant protein disequilibrium. Although the molecules implicated in distinct organisms are not necessarily the same, it seems that alternatives to regulate proteaphagy are multiple. The way in which proteaphagy could impact other proteasome complexes and proteasome-regulated events in distinct cellular compartments, such as the nucleus, remains to be investigated. Finally, proteaphagy can help cells to survive stresses like nutrient starvation, since proteasomes work with a high energy cost and represent a potential source of amino acid for recycling. Unravelling the regulation and biological impact of proteaphagy could potentially have clinical implications, as new evidence underlines an important dysregulation of this mechanism in various human pathologies.

## Figures and Tables

**Table 1:** Structure and functions associated to the most studied autophagy receptors. Among more than 30 autophagy receptors known so far in mammalians, structural similarities and preserved functional domains are observed such as the presence of ubiquitin binding domains (UBDs), ATG8s binding domains like LC3 interacting regions (LIRs), oligomerization domains like PB1, or membrane associated domain [65]. Involvement of the displayed autophagy receptors in distinct selective autophagy events and collaborations between them are listed.

**Autophagy Receptor**	**Structure** 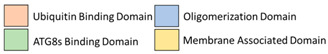	**Selective Autophagy**	**Collaboration**	**References**
p62/SQSTM1	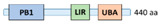	Aggrephagy; Mitophagy; Xenophagy; Lysophagy; Pexophagy; Proteaphagy	NBR1 (aggregaphagy, pexophagy) NDP52 + OPTN (xenophagy)	[66,67,68]
NBR1	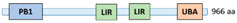	Pexophagy; Aggrephagy	p62 (aggregaphagy, pexophagy);	[64,66,67]
NDP52	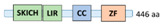	Mitophagy	p62 + OPTN (xenophagy)	[67,69]
OPTN	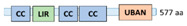	Mitophagy; Xenophagy	NDP52 + p62 (xenophagy)	[67,69,70]
BNIP3/NIX	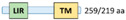	Mitophagy		[71]
ALFY	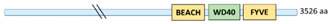	Aggrephagy	p62 (aggrephagy)	[72,73]
RTN3	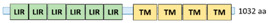	ER-phagy		[74]
FAM134B	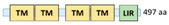	ER-phagy; Aggrephagy		[75]
